# Supplementary material for: Regulation associated modules reflect 3D genome modularity associated with chromatin activity
Source: Nat Commun. 2022 Sep 8;13:5281. doi: 10.1038/s41467-022-32911-y (PMC9458634; doi:10.1038/s41467-022-32911-y)
Supplement: Supplementary file 1 — Supplementary Information [file 41467_2022_32911_MOESM1_ESM.pdf]

Supplementary Information

Regulation associated modules reflect 3D genome modularity associated with chromatin activity

Lina Zheng[1], Wei Wang[1,2,3,*]

[1] Bioinformatics and Systems Biology Program, University of California San Diego, La Jolla, CA 92093-0359.

[2] Department of Chemistry and Biochemistry, University of California San Diego, La Jolla, CA 92093-0359.

[3] Department of Cellular and Molecular Medicine, University of California San Diego, La Jolla, CA 92093-0359.

[*] Correspondence: wei-wang@ucsd.edu

This PDF file includes:

Supplementary Figures 1-7
Supplementary Tables 1- 5

Supplementary Figures:

Supplementary Figure 1. The frequency of the consensus RAM boundaries in the 93 normal samples.

Supplementary Figure 2. The frequency of the consensus RAM boundaries in the 19 cancer samples.

Supplementary Figure 3. Consensus RAMs.

Supplementary Figure 4. RAMs are defined at a scale better aligned with chromatin activities.

Supplementary Figure 5. RAMs are resistant to cohesin degradation.

Supplementary Figure 6. Predicted chromatin structure change upon deletion of cRAM boundaries and TAD boundaries in H1-hESC cell line.

Supplementary Figure 7. Gene Ontology Molecular Functions of the genes within 2.5kb from the enriched motifs overlapping with somatic indels.

Supplementary Tables:

Supplementary Table 1. Collected ChIP-seq samples from ROADMAP and ENCODE.

Supplementary Table 2. Upregulated gene expressions involved in enhancer-promoter interactions occurred in the same RAM in K562 but in different GM12878 RAMs.

Supplementary Table 3. Upregulated gene expressions involved in enhancer-promoter interactions occurred in the same RAM in K562 but in different HEPG2 RAMs.

Supplementary Table 4. Essential non-coding loci in cancer cRAM boundaries.

Supplementary Table 5. DEGs annotated with Gene Ontology Molecular Function of "Calcium Ion Binding".

Supplementary Figures

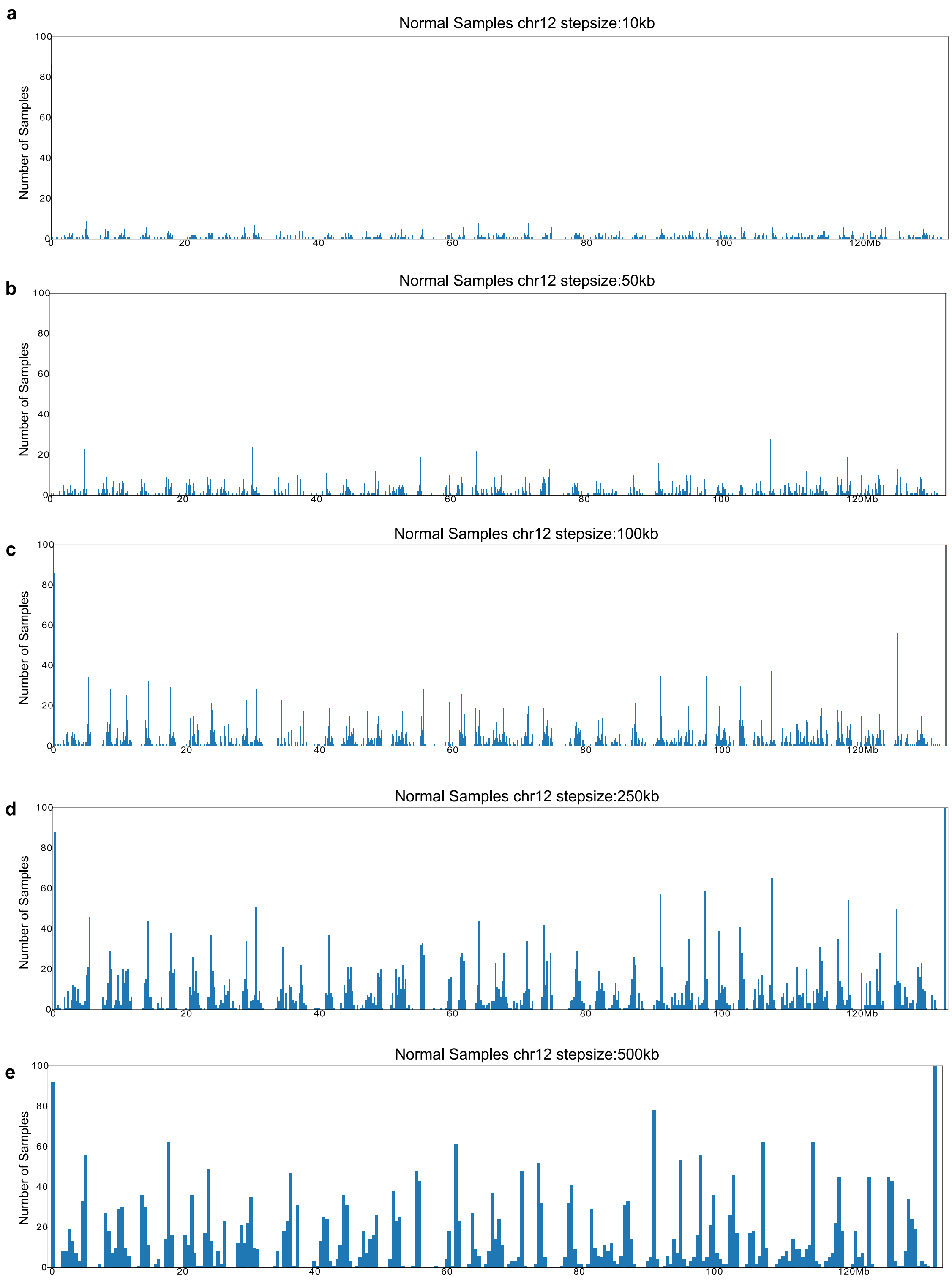

Supplementary Figure 1. The frequency of the consensus RAM boundaries in the 93 normal samples. The step sizes are **a** 10kb, **b** 50kb, **c**100kb, **d** 250kb, **e** 500kb.

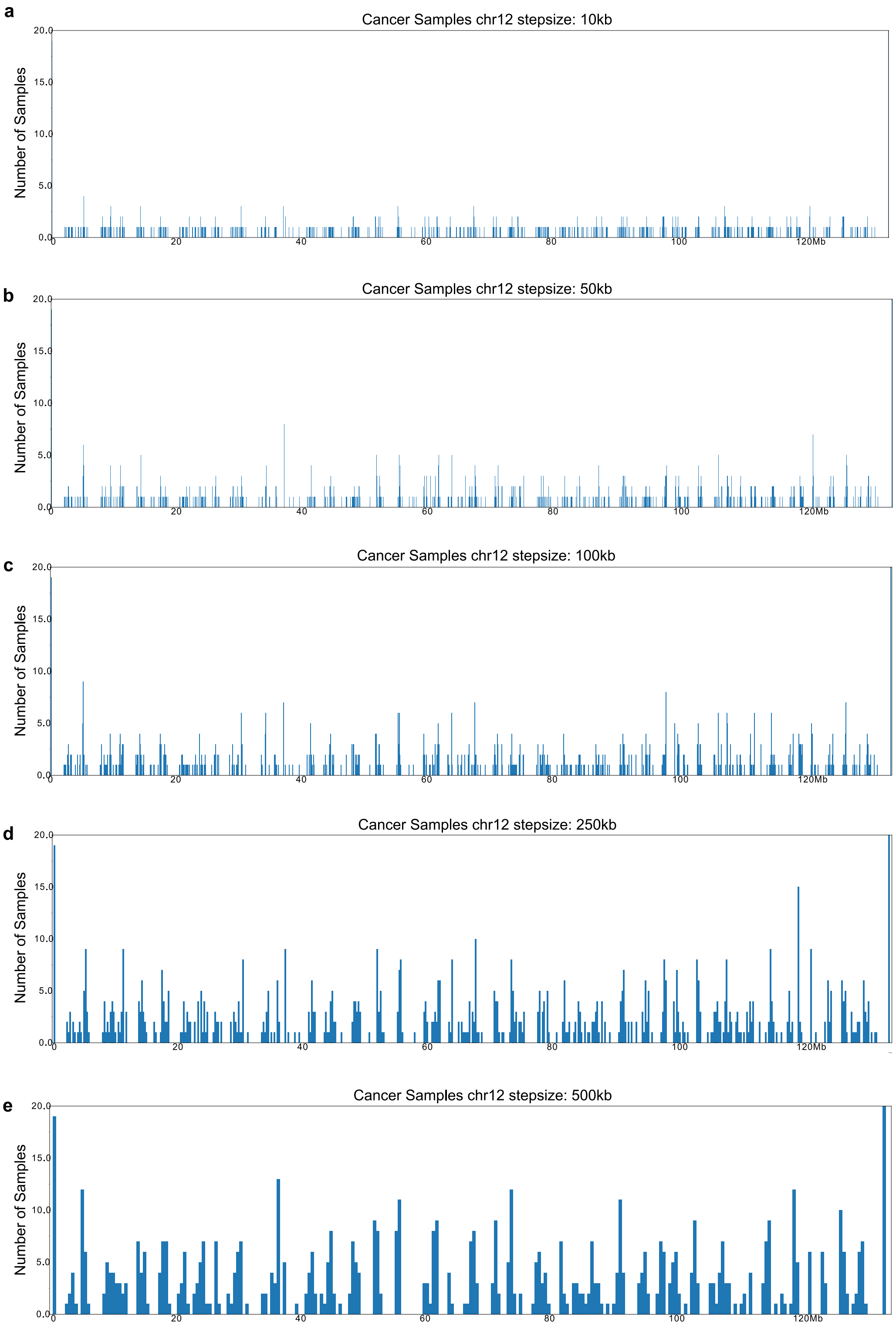

Supplementary Figure 2. The frequency of the consensus RAM boundaries in the 19 cancer samples. The step sizes are **a** 10kb, **b** 50kb, **c**100kb, **d** 250kb, **e** 500kb.

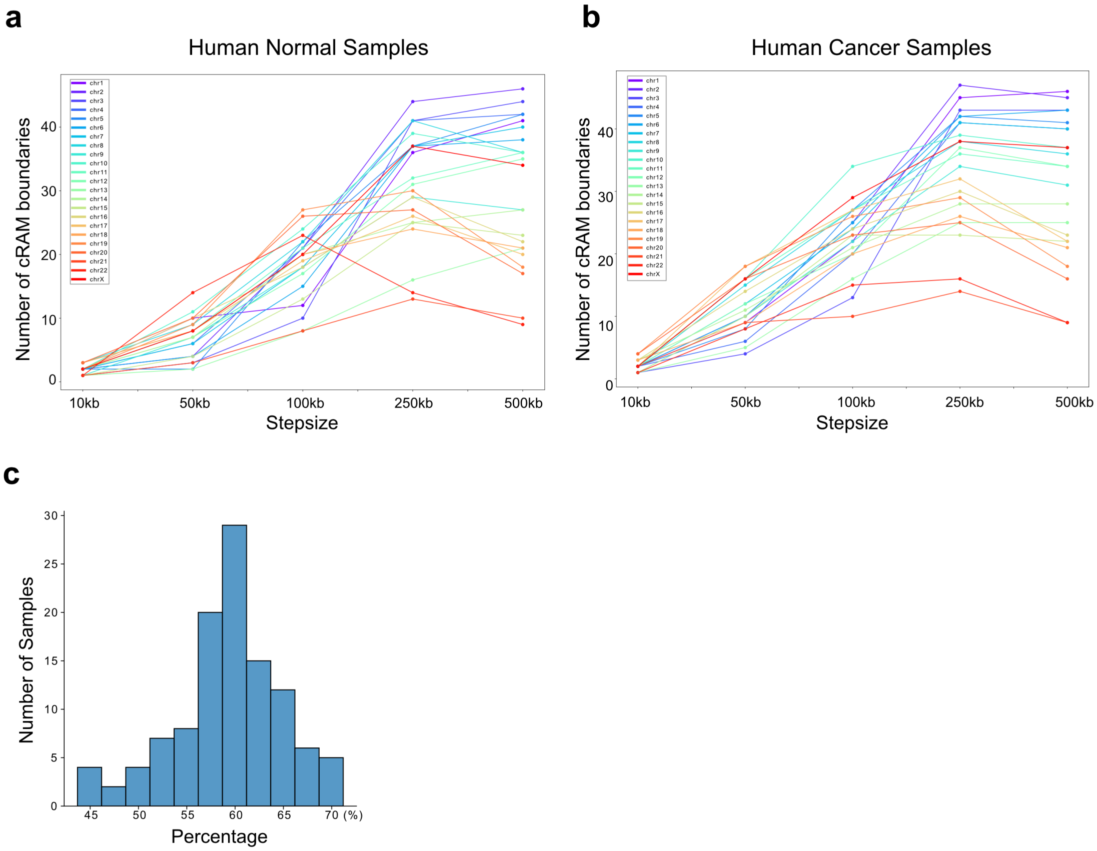

Supplementary Figure 3. Consensus RAMs. The number of consensus RAMs (i.e. RAMs shared between samples) using different step sizes in **a** normal, **b** cancer samples. **c** The percentage of the consensus RAMs among all the RAMs in each sample.

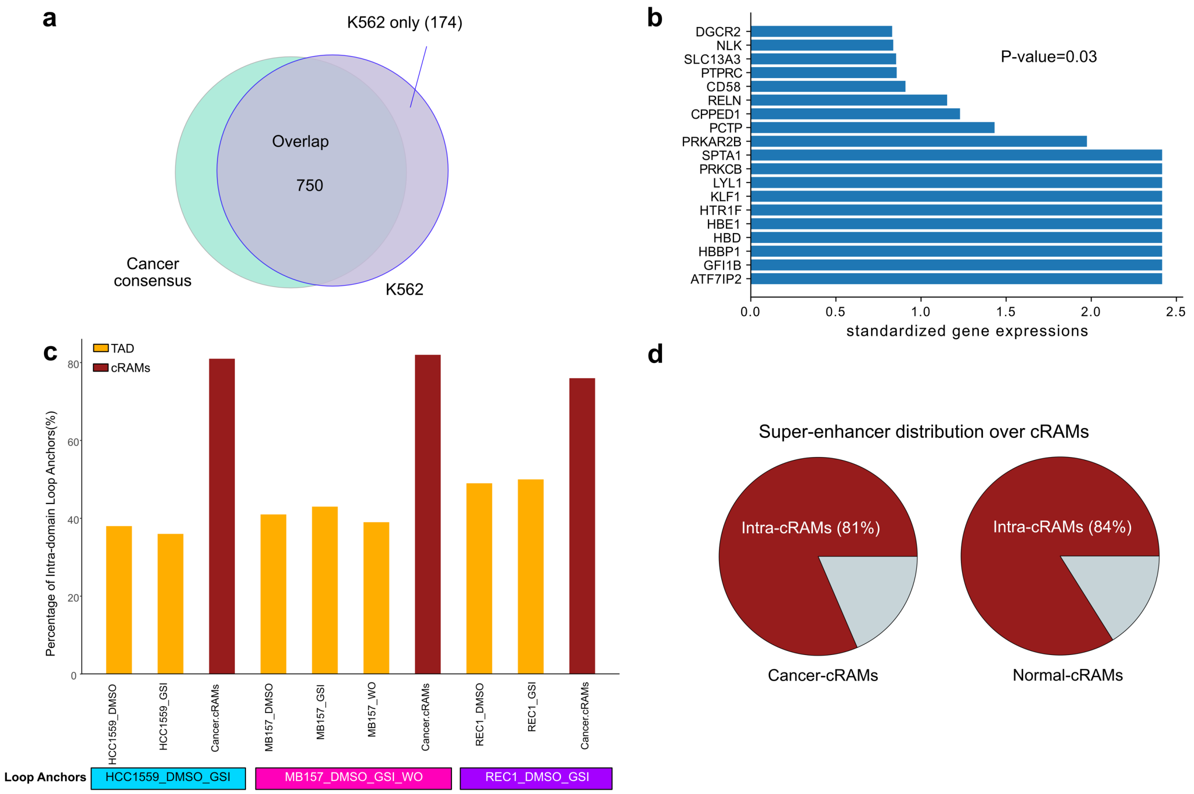

Supplementary Figure 4. RAMs are defined at a scale better aligned with chromatin activities. a K562 enhancer-promoter pairs distribution over K562 RAMs and cancer cRAMs. **b** 19 highly expressed genes in the enhancer-promoter pairs were uniquely observed in K562 RAMs. The p-value was derived from hypergeometric test. **c** Percentage of intra-domain loop anchors in TADs and cRAMs. **d** Super-enhancer distribution within the same cRAMs.

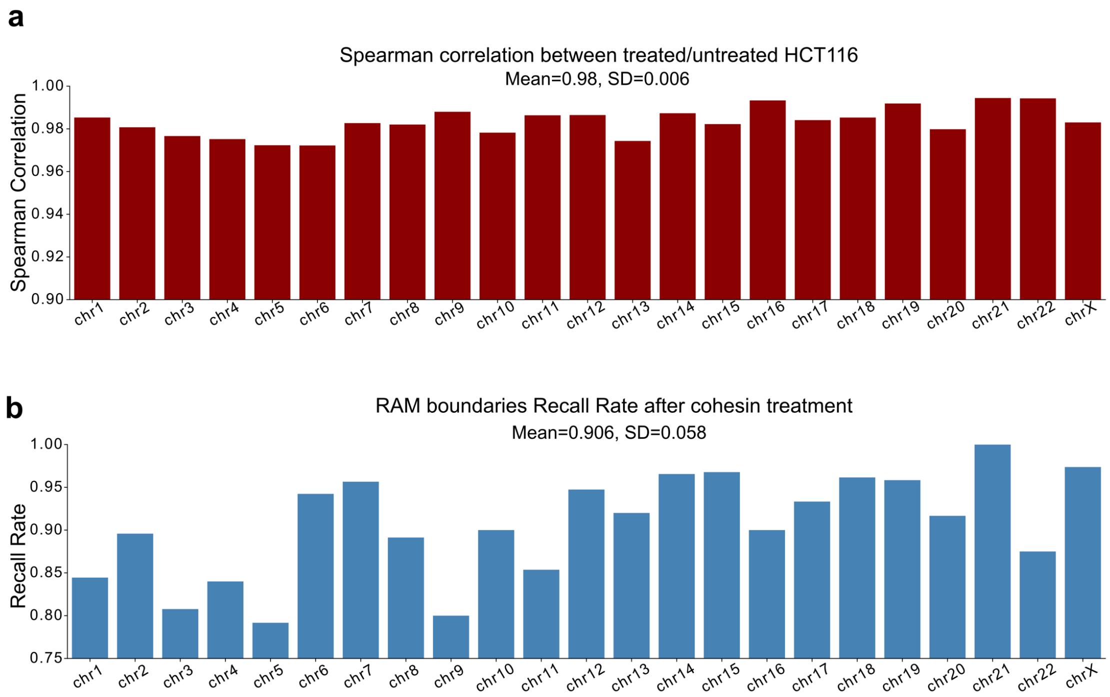

Supplementary Figure 5. RAMs are resistant to cohesin degradation. a Spearman correlation of the RAMs between the treated and untreated HCT116 cells. **b** RAM boundaries recall rate after cohesin treatment for HCT116 cells.

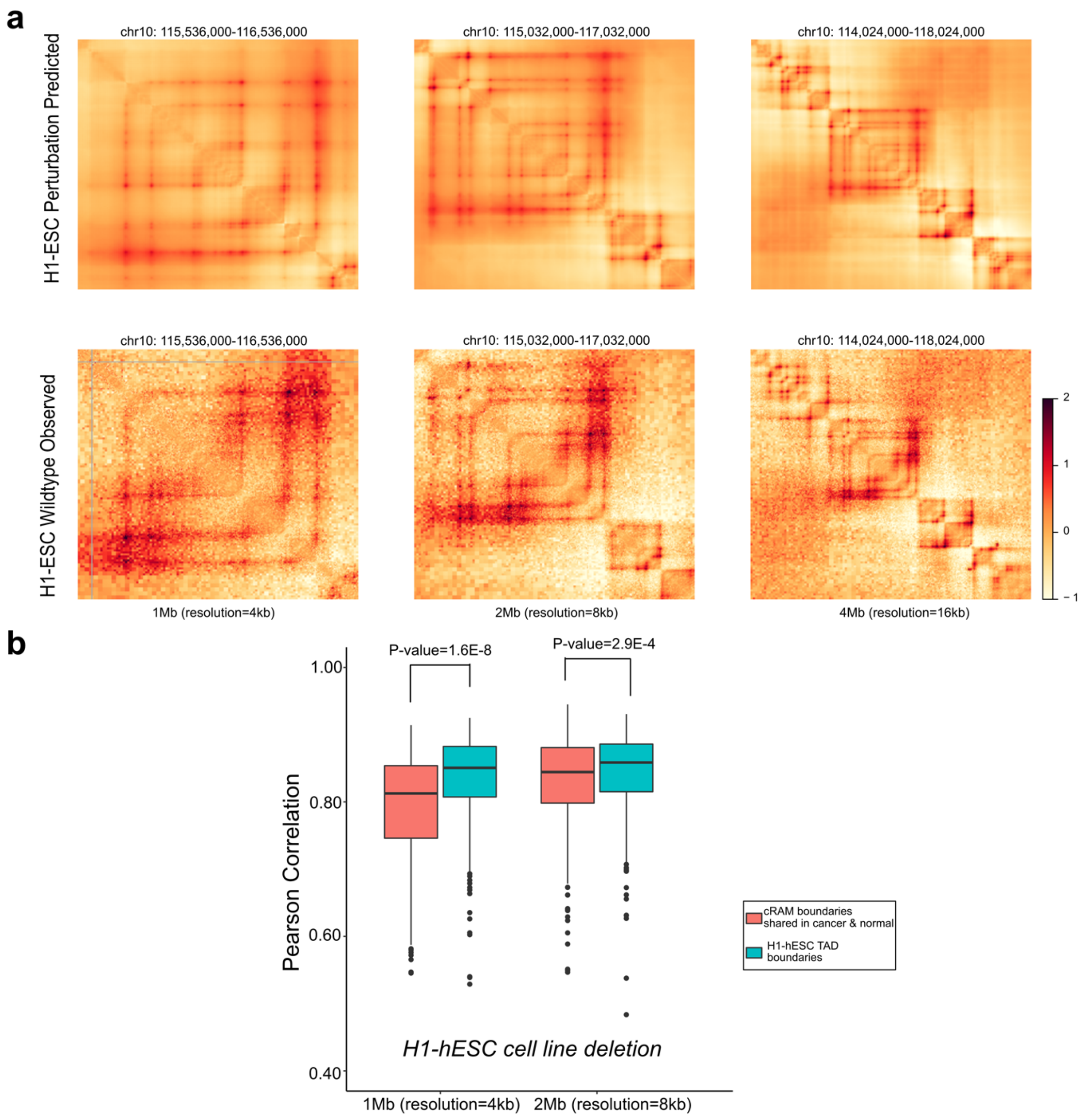

Supplementary Figure 6. Predicted chromatin structure change upon deletion of cRAM boundaries and TAD boundaries in H1-hESC cell line. a An example of the Hi-C contact change upon deletion of the cRAM boundary (chr10:115,940,000-116,040,000 in hg38) in H1-hESC cells predicted by a deep learning model ORCA. **b** Pearson correlations between the predicted Hi-C contacts before and after cRAM boundary and TAD boundary deletion in H1-hESC cells. A lower correlation indicates a larger perturbation to the wildtype chromatin structure upon deletion. n=427 loci in 1Mb resolution and n=1177 loci in 2Mb resolution data for H1 cell line. The p-value was derived from two-sided Wilcoxon Rank Sum. The bounds of all boxplots showed the 25 percentile,

median, 75 percentile of the dataset; the maxima and minima were defined excluding the outliers.

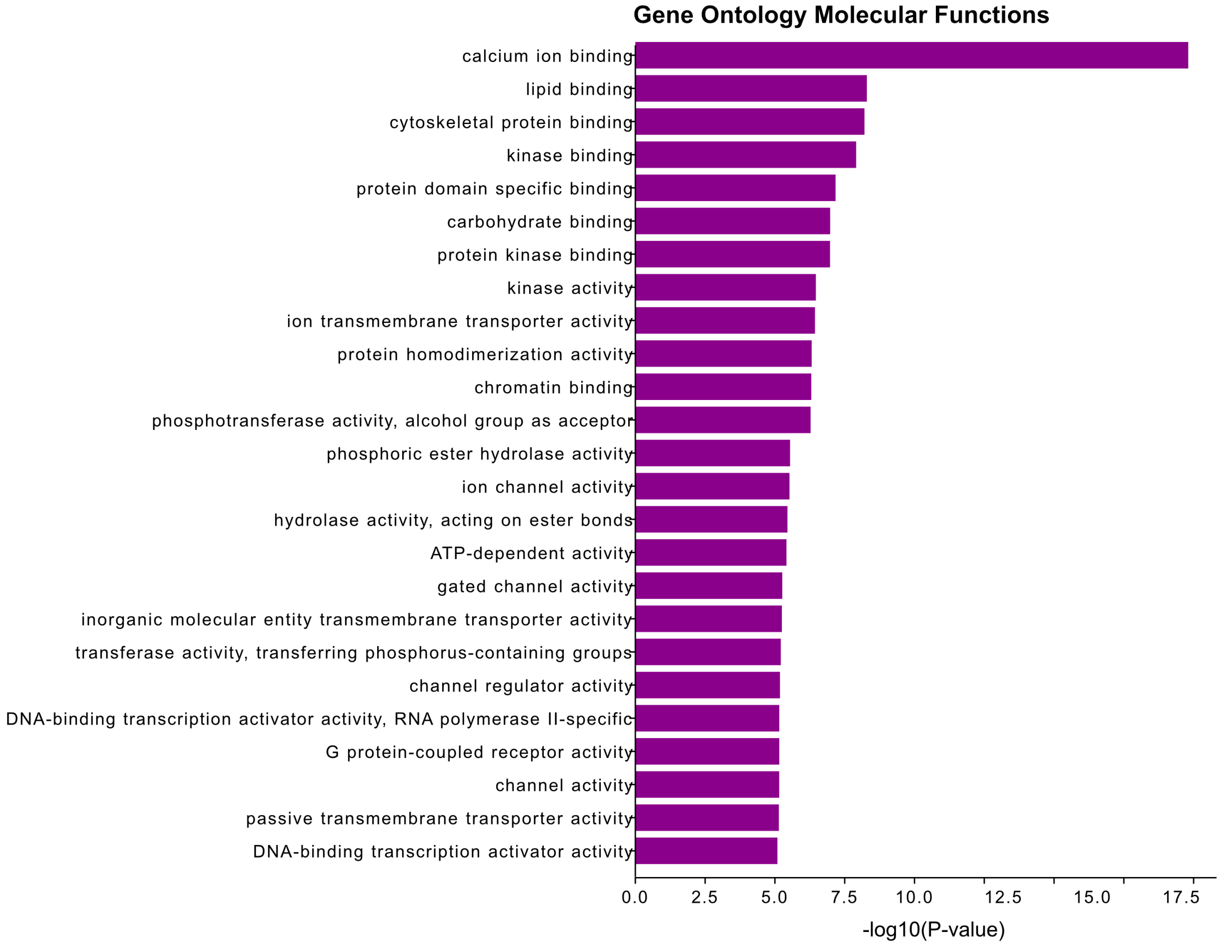

Supplementary Figure 7. Gene Ontology Molecular Functions of the genes within 2.5kb from the enriched motifs overlapping with somatic indels. The p-value was derived from hypergeometric test with Benjamini–Hochberg False Discovery Rate approach at FDR cutoff 0.05 in g:Profiler software.

Supplementary Tables

Supplementary Table 1. Collected ChIP-seq samples from ROADMAP and ENCODE.

Data ID	Source	Tissue/SampleType	Details
E017	Roadmap	IMR90	IMR90 fetal lung fibroblasts Cell Line
E008	Roadmap	ESC	H9 Cells
E015	Roadmap	ESC	HUES6 Cells
E014	Roadmap	ESC	HUES48 Cells
E016	Roadmap	ESC	HUES64 Cells
E003	Roadmap	ESC	H1 Cells
E020	Roadmap	iPSC	iPS-20b Cells
E019	Roadmap	iPSC	iPS-18 Cells
E021	Roadmap	iPSC	iPS DF 6.9 Cells
E022	Roadmap	iPSC	iPS DF 19.11 Cells
E007	Roadmap	ES-deriv	H1 Derived Neuronal Progenitor Cultured Cells
E013	Roadmap	ES-deriv	hESC Derived CD56+ Mesoderm Cultured Cells
E012	Roadmap	ES-deriv	hESC Derived CD56+ Ectoderm Cultured Cells
E011	Roadmap	ES-deriv	hESC Derived CD184+ Endoderm Cultured Cells
E004	Roadmap	ES-deriv	H1 BMP4 Derived Mesendoderm Cultured Cells
E005	Roadmap	ES-deriv	H1 BMP4 Derived Trophoblast Cultured Cells
E006	Roadmap	ES-deriv	H1 Derived Mesenchymal Stem Cells
E062	Roadmap	Blood&Tcell	Primary mononuclear cells from peripheral blood
E034	Roadmap	Blood&Tcell	Primary T cells from peripheral blood
E045	Roadmap	Blood&Tcell	Primary T cells effector/memory enriched from peripheral blood
E044	Roadmap	Blood&Tcell	Primary T regulatory cells from peripheral blood
E043	Roadmap	Blood&Tcell	Primary T helper cells from peripheral blood
E039	Roadmap	Blood&Tcell	Primary T helper naive cells from peripheral blood
E041	Roadmap	Blood&Tcell	Primary T helper cells PMA-I stimulated
E042	Roadmap	Blood&Tcell	Primary T helper 17 cells PMA-I stimulated
E040	Roadmap	Blood&Tcell	Primary T helper memory cells from peripheral blood 1
E037	Roadmap	Blood&Tcell	Primary T helper memory cells from peripheral blood 2
E048	Roadmap	Blood&Tcell	Primary T CD8+ memory cells from peripheral blood
E038	Roadmap	Blood&Tcell	Primary T helper naive cells from peripheral blood
E047	Roadmap	Blood&Tcell	Primary T CD8+ naive cells from peripheral blood
E029	Roadmap	HSC&Bcell	Primary monocytes from peripheral blood
E050	Roadmap	HSC&Bcell	Primary hematopoietic stem cells G-CSF-mobilized Female

E032	Roadmap	HSC&Bcell	Primary B cells from peripheral blood
E046	Roadmap	HSC&Bcell	Primary Natural Killer cells from peripheral blood
E026	Roadmap	Mesench	Bone Marrow Derived Cultured Mesenchymal Stem Cells
E049	Roadmap	Mesench	Mesenchymal Stem Cell Derived Chondrocyte Cultured Cells
E055	Roadmap	Epithelial	Foreskin Fibroblast Primary Cells skin01
E056	Roadmap	Epithelial	Foreskin Fibroblast Primary Cells skin02
E059	Roadmap	Epithelial	Foreskin Melanocyte Primary Cells skin01
E061	Roadmap	Epithelial	Foreskin Melanocyte Primary Cells skin03
E058	Roadmap	Epithelial	Foreskin Keratinocyte Primary Cells skin03
E112	Roadmap	Thymus	Thymus
E093	Roadmap	Thymus	Fetal Thymus
E071	Roadmap	Brain	Brain Hippocampus Middle
E074	Roadmap	Brain	Brain Substantia Nigra
E068	Roadmap	Brain	Brain Anterior Caudate
E069	Roadmap	Brain	Brain Cingulate Gyrus
E072	Roadmap	Brain	Brain Inferior Temporal Lobe
E067	Roadmap	Brain	Brain Angular Gyrus
E073	Roadmap	Brain	Brain_Dorsolateral_Prefrontal_Cortex
E063	Roadmap	Adipose	Adipose Nuclei
E100	Roadmap	Muscle	Psoas Muscle
E108	Roadmap	Muscle	Skeletal Muscle Female
E089	Roadmap	Muscle	Fetal Muscle Trunk
E090	Roadmap	Muscle	Fetal Muscle Leg
E104	Roadmap	Heart	Right Atrium
E095	Roadmap	Heart	Left Ventricle
E105	Roadmap	Heart	Right Ventricle
E065	Roadmap	Heart	Aorta
E078	Roadmap	Sm. Muscle	Duodenum Smooth Muscle
E076	Roadmap	Sm. Muscle	Colon Smooth Muscle
E103	Roadmap	Sm. Muscle	Rectal Smooth Muscle
E111	Roadmap	Sm. Muscle	Stomach Smooth Muscle
E092	Roadmap	Digestive	Fetal Stomach
E085	Roadmap	Digestive	Fetal Intestine Small
E084	Roadmap	Digestive	Fetal Intestine Large
E109	Roadmap	Digestive	Small Intestine
E106	Roadmap	Digestive	Sigmoid Colon

E075	Roadmap	Digestive	Colonic Mucosa
E101	Roadmap	Digestive	Rectal Mucosa Donor 29
E102	Roadmap	Digestive	Rectal Mucosa Donor 31
E079	Roadmap	Digestive	Esophagus
E094	Roadmap	Digestive	Gastric
E099	Roadmap	PLCNT.AMN	Placenta Amnion
E097	Roadmap	OVRY	Ovary
E087	Roadmap	PANC.ISLT	Pancreatic Islets
E080	Roadmap	ADRL.GLND.FET	Fetal Adrenal Gland
E091	Roadmap	PLCNT.FET	Placenta
E066	Roadmap	LIV.ADLT	Liver
E098	Roadmap	PANC	Pancreas
E096	Roadmap	LNG	Lung
E113	Roadmap	SPLN	Spleen
E116	Roadmap	BLD.GM12878	GM12878 Lymphoblastoid Cells
E119	Roadmap	BRST.HMEC	HMEC Mammary Epithelial Primary Cells
E120	Roadmap	MUS.HSMM	HSMM Skeletal Muscle Myoblasts Cells
E121	Roadmap	MUS.HSMMT	HSMM cell derived Skeletal Muscle Myotubes Cells
E122	Roadmap	VAS.HUVEC	HUVEC Umbilical Vein Endothelial Primary Cells
E124	Roadmap	BLD.CD14.MONO	Monocytes-CD14+ RO01746 Primary Cells
E125	Roadmap	BRN.NHA	NH-A Astrocytes Primary Cells
E126	Roadmap	SKIN.NHDFAD	NHDF-Ad Adult Dermal Fibroblast Primary Cells
E127	Roadmap	SKIN.NHEK	NHEK-Epidermal Keratinocyte Primary Cells
E128	Roadmap	LNG.NHLF	NHLF Lung Fibroblast Primary Cells
E129	Roadmap	BONE.OSTEO	Osteoblast Primary Cells
E114	Roadmap	LNG.A549.ETOH002.CNCR	A549 EtOH 0.02pct Lung Carcinoma Cell Line
E115	Roadmap	BLD.DND41.CNCR	Dnd41 TCell Leukemia Cell Line
E117	Roadmap	CRVX.HELAS3.CNCR	HeLa-S3 Cervical Carcinoma Cell Line
E118	Roadmap	LIV.HEPG2.CNCR	HepG2 Hepatocellular Carcinoma Cell Line
E123	Roadmap	BLD.K562.CNCR	K562 Leukemia Cells
ENCFF409EFR	Encode	SK-N-MC	neuroblastoma
ENCFF575WAS	Encode	HCT116	colon
ENCFF209VEY	Encode	PC-3	prostatic
ENCFF831KZM	Encode	MCF-7	Breast
ENCFF787ITI	Encode	OCI-LY3	non-Hodgkin.lymphoma
ENCFF161GCD	Encode	ACC112	Adenoid.cystic.carcinoma

ENCFF468GKP	Encode	SK-N-SH	neuroblastoma
ENCFF137AXJ	Encode	VCaP	prostate
ENCFF629BRY	Encode	Panc1	pancreatic
ENCFF159JKE	Encode	C4-2B	HPV.cervical
ENCFF623PRE	Encode	22Rv1	prostate
ENCFF279PSG	Encode	OCI-LY1	non-Hodgkin.lymphoma
ENCFF262PTI	Encode	A673	rhabdomyosarcoma
ENCFF152UAP	Encode	PC-9	lung

Supplementary Table 2. Upregulated gene expressions involved in enhancer-promoter interactions occurred in the same RAM in K562 but in different GM12878 RAMs.

Genes	log2(GM12878)	log2(K562)	Difference log2(K562/GM12878)	Foldchange
PTGER3	0.01	1.77	1.76	3.38
CLTCL1	0.05	2.65	2.60	6.07
DGCR2	2.56	4.90	2.34	5.06
HDAC6	1.96	4.27	2.31	4.95
PLP2	5.57	7.04	1.47	2.77
RHAG	0.03	9.71	9.68	820.61
PFKFB4	5.10	5.14	0.04	1.03
HPCAL1	1.93	3.65	1.72	3.29
ARL4A	0.09	6.03	5.93	61.15
GIPC1	2.81	4.37	1.56	2.94
VGF	0.00	0.24	0.24	1.18
APIP	2.74	3.62	0.88	1.84
RAB31	0.55	5.65	5.10	34.29
HTR1F	0.01	1.47	1.46	2.75
MEX3B	1.09	2.85	1.76	3.39
PRELID2	0.13	2.56	2.42	5.37
MITF	0.48	2.53	2.05	4.14

Supplementary Table 3. Upregulated gene expressions involved in enhancer-promoter interactions occurred in the same RAM in K562 but in different HEPG2 RAMs.

Genes	log2(HEPG2)	log2(K562)	Difference log2(K562/HEPG2)	FoldChange
ZNF582	0.63	1.34	0.71	1.64
PTGER3	0.01	1.77	1.76	3.39
CLTCL1	2.30	2.65	0.36	1.28
PTPRC	0.00	3.00	3.00	7.99
RNF24	3.26	4.82	1.56	2.95
CTSC	5.01	5.10	0.10	1.07
KAT2B	1.29	3.23	1.94	3.85
TPST2	4.07	6.17	2.09	4.27
CCDC74A	0.06	3.20	3.14	8.83
ZEB2	0.01	4.35	4.34	20.20
SNX18	1.59	2.32	0.74	1.66
HTR1F	0.00	1.47	1.47	2.76
MEX3B	0.68	2.85	2.17	4.51
ZNF431	1.80	4.12	2.32	4.98
CD47	0.86	3.75	2.89	7.40

Supplementary Table 4. Essential non-coding loci in cancer cRAM boundaries.

chromosome	Start	End	Essential Non-coding locus Name
chr10	115000	119999	Hub_10_1
chr16	29195000	29199999	Hub_16_12
chr16	29270000	29274999	Hub_16_22
chr16	88270000	88274999	Hub_16_73
chr21	15240000	15244999	Hub_21_23
chr6	62315000	62319999	Hub_6_36
chr7	4480000	4484999	Hub_7_1
chr7	9360000	9364999	Hub_7_9
chr8	47000000	47004999	Hub_8_24
chr5	161700000	161704999	Hub_5_36
chr8	47060000	47064999	Hub_8_25
chr8	46925000	46929999	Hub_8_21
chr7	46365000	46369999	Hub_7_49
chr1	106485000	106489999	Hub_1_19

Supplementary Table 5. DEGs annotated with Gene Ontology Molecular Function of "Calcium Ion Binding". P-value was derived from two-sided Wilcoxon Rank Sum Test.

Gene	Cancer_mean	Normal_mean	log2(Cancer/Nromal)	P-value
AIF1L	7.86	25.48	-1.70	1.40E-02
CABP1	0.16	4.40	-4.79	6.66E-06
CDH1	49.64	24.00	1.05	4.46E-03
CDH3	16.24	5.80	1.49	9.34E-03
DGKB	0.23	1.16	-2.34	1.62E-03
FAT3	0.31	0.56	-0.86	9.49E-03
HMCN2	0.24	1.43	-2.54	4.03E-03
ITSN1	1.64	2.70	-0.72	4.11E-04
LRP1B	0.20	0.57	-1.49	1.45E-03
MAN1C1	2.14	3.60	-0.75	7.23E-04
MASP1	1.31	3.35	-1.36	5.79E-05
MCTP1	0.64	1.39	-1.12	4.30E-02
MYL3	0.41	8.36	-4.33	2.63E-09
NCS1	7.01	37.53	-2.42	2.37E-03
NDUFAB1	30.63	22.33	0.46	2.93E-05
PCDHA10	0.06	0.17	-1.39	1.09E-02
PCDHA2	0.03	0.08	-1.18	3.57E-02
PCDHA3	0.06	0.14	-1.25	3.82E-03
PCDHA6	0.01	0.10	-2.69	2.64E-03
RYR2	0.13	1.73	-3.78	7.88E-07
SELL	10.52	17.24	-0.71	1.12E-04
SVEP1	0.84	3.08	-1.87	2.39E-04
SYT2	0.07	1.16	-4.12	9.57E-07
VSNL1	2.05	26.51	-3.69	3.34E-02
WDR49	0.10	0.35	-1.86	2.87E-02
