# Supplementary material for: Regulation associated modules reflect 3D genome modularity associated with chromatin activity
Source: Nat Commun. 2022 Sep 8;13:5281. doi: 10.1038/s41467-022-32911-y (PMC9458634; doi:10.1038/s41467-022-32911-y)
Supplement: Supplementary file 2 — Reporting Summary [file 41467_2022_32911_MOESM2_ESM.pdf]

nature portfolio

Corresponding author(s): Wei Wang

Last updated by author(s): 8/7/2022

Reporting Summary

Nature Portfolio wishes to improve the reproducibility of the work that we publish. This form provides structure for consistency and transparency in reporting. For further information on Nature Portfolio policies, see our Editorial Policies and the Editorial Policy Checklist.

Statistics

For all statistical analyses, confirm that the following items are present in the figure legend, table legend, main text, or Methods section.

n/a	Confirmed	
☐	☒	The exact sample size (*n*) for each experimental group/condition, given as a discrete number and unit of measurement
☐	☒	A statement on whether measurements were taken from distinct samples or whether the same sample was measured repeatedly
☐	☒	The statistical test(s) used AND whether they are one- or two-sided<br>*Only common tests should be described solely by name; describe more complex techniques in the Methods section.*
☒	☐	A description of all covariates tested
☐	☒	A description of any assumptions or corrections, such as tests of normality and adjustment for multiple comparisons
☐	☒	A full description of the statistical parameters including central tendency (e.g. means) or other basic estimates (e.g. regression coefficient) AND variation (e.g. standard deviation) or associated estimates of uncertainty (e.g. confidence intervals)
☐	☒	For null hypothesis testing, the test statistic (e.g. *F*, *t*, *r*) with confidence intervals, effect sizes, degrees of freedom and *P* value noted<br>*Give P values as exact values whenever suitable.*
☒	☐	For Bayesian analysis, information on the choice of priors and Markov chain Monte Carlo settings
☒	☐	For hierarchical and complex designs, identification of the appropriate level for tests and full reporting of outcomes
☐	☒	Estimates of effect sizes (e.g. Cohen's *d*, Pearson's *r*), indicating how they were calculated

Our web collection on statistics for biologists contains articles on many of the points above.

Software and code

Policy information about availability of computer code

Data collection	GNU wget version 1.14
Data analysis	R (version 4.1.2); Python (version 3.8); Juicer pipeline (version 1.5.6); BWA (version 0.7.17); Picard (version 2.17.0); MACS2 (version 2.2.7.1); bedops (version 2.4.40); Homer (version 4.11.1); MEME Suite (version 5.4.1); HiCPlotter (version 0.8.1); HiCExplorer (version 2.2.1.1)

For manuscripts utilizing custom algorithms or software that are central to the research but not yet described in published literature, software must be made available to editors and reviewers. We strongly encourage code deposition in a community repository (e.g. GitHub). See the Nature Portfolio guidelines for submitting code & software for further information.

Data

Policy information about availability of data

All manuscripts must include a data availability statement. This statement should provide the following information, where applicable:

- Accession codes, unique identifiers, or web links for publicly available datasets
- A description of any restrictions on data availability
- For clinical datasets or third party data, please ensure that the statement adheres to our policy

All the data to evaluate the conclusions during this study are included in the article and its supplementary information files. The 93 normal and 19 cancer samples with processed narrow peaks (H3K27ac, H3K4me3, H3K4me1) and broad peaks (H3K27me3, H3K9me3, H3K36me3) in hg19 were downloaded from Roadmap Epigenomics portal [https://egg2.wustl.edu/roadmap/web_portal/] and ENCODE portal [https://www.encodeproject.org/]. The Hi-C data for the wildtype K562, GM12878, A549, IMR90, NHEK, HUVEC, HMEC, HCT116 and HFF cell lines were downloaded from GSE63525 [https://www.ncbi.nlm.nih.gov/geo/query/acc.cgi?

acc=GSE63525], ENCSR662QKG [https://www.encodeproject.org/experiments/ENCSR662QKG/], GSE104333 [https://www.ncbi.nlm.nih.gov/geo/query/acc.cgi?acc=GSE104333], and 4DNFIMROE6N4 [https://data.4dnucleome.org/files-processed/4DNFIMROE6N4/]. The H3K27ac ChIP-seq data in the untreated HCT-116 RAD21-mAC cells and HCT-116 RAD21-mAC cells treated for 6 hours with IAA were downloaded from GSE104888 [https://www.ncbi.nlm.nih.gov/geo/query/acc.cgi?acc=GSE104888]. The consensus somatic SNV and indels were downloaded from PCAWG [https://dcc.icgc.org/releases/PCAWG]. The K562, HCT116, H1, HAP1, RPE-hTERT, HFFc6 cell lines LAD data were downloaded through 4DNFIJHD22QE [https://data.4dnucleome.org/files-processed/4DNFIJHD22QE/], 4DNFIA2LBQCD [https://data.4dnucleome.org/files-processed/4DNFIA2LBQCD/], 4DNFIJXADI29 [https://data.4dnucleome.org/files-processed/4DNFIJXADI29/], 4DNFIDFCY3JN [https://data.4dnucleome.org/files-processed/4DNFIDFCY3JN/], 4DNFIUVTO2H3 [https://data.4dnucleome.org/files-processed/4DNFIUVTO2H3/], 4DNFIT9W77EE [https://data.4dnucleome.org/files-processed/4DNFIT9W77EE/].

Human research participants

Policy information about studies involving human research participants and Sex and Gender in Research.

Reporting on sex and gender	Not Applicable
Population characteristics	Not Applicable
Recruitment	Not Applicable
Ethics oversight	Not Applicable

Note that full information on the approval of the study protocol must also be provided in the manuscript.

Field-specific reporting

Please select the one below that is the best fit for your research. If you are not sure, read the appropriate sections before making your selection.

[x] Life sciences   [ ] Behavioural & social sciences   [ ] Ecological, evolutionary & environmental sciences

For a reference copy of the document with all sections, see nature.com/documents/nr-reporting-summary-flat.pdf

Life sciences study design

All studies must disclose on these points even when the disclosure is negative.

Sample size	Not Applicable. We collected all the publicly available data. The statistics tests are derived from genomic loci comparison, which are usually above 30 loci in all the samples. So the sample size is sufficient to support our conclusion.
Data exclusions	No data exclusion
Replication	Not Applicable. We collected all the publicly available data, no specifically designed replication.
Randomization	Not Applicable. We collected all the publicly available data, no specifically designed randomization.
Blinding	Not Applicable. We collected all the publicly available data, no specifically designed blinding.

Reporting for specific materials, systems and methods

We require information from authors about some types of materials, experimental systems and methods used in many studies. Here, indicate whether each material, system or method listed is relevant to your study. If you are not sure if a list item applies to your research, read the appropriate section before selecting a response.

Materials & experimental systems

n/a	Involved in the study	
[x]	[ ]	Antibodies
[x]	[ ]	Eukaryotic cell lines
[x]	[ ]	Palaeontology and archaeology
[x]	[ ]	Animals and other organisms
[x]	[ ]	Clinical data
[x]	[ ]	Dual use research of concern

Methods

n/a	Involved in the study	
[x]	[ ]	ChIP-seq
[x]	[ ]	Flow cytometry
[x]	[ ]	MRI-based neuroimaging
